# Human Intestinal Parasites: Prevalence and Associated Risk Factors among Grade School Children in Maksegnit, Northwest Ethiopia

**DOI:** 10.1155/2021/6694809

**Published:** 2021-06-10

**Authors:** Kefale Shiferaw, Teklemichael Tesfay, Girmay Kalayu, Gebrehiwot Kiros

**Affiliations:** Aksum University, College of Natural and Computational Science, Department of Biology, Aksum, Ethiopia

## Abstract

This study was aimed to assess the prevalence and associated risk factors of intestinal parasitic infections in grade school children in Maksegnit, Northwest Ethiopia. Five species of intestinal parasites were identified with an overall prevalence of 155 (40.4%). Among these, *Ascaris lumbricoides* 122 (31.8%) and *Entamoeba histolytica* 18 (4.7%) were predominant. Of the total 155 (40.4%) positive individuals, 149 (39%) had a single infection and the rest 6 (1.6%) had double parasitic infections. Of the different variables assessed, age, gender, shoe wearing, and eating raw or undercooked vegetables were not significantly associated with the prevalence of intestinal parasites (*P* > 0.05). However, a statistically significant association (*P* < 0.05) was observed between infected children and variables including defecation habit (AOR = 0.216), cleanliness of fingernails (AOR = 0.146), drinking river water (AOR = 0.124), and hand washing habit after defecation (AOR = 0.236) (*P* < 0.05). Regular deworming, education on personal hygiene, and environmental sanitation to both students and their parents shall be implemented to reduce the prevalence rate of intestinal parasitic infections in the study area.

## 1. Introduction

Intestinal parasitic infections (IPIs) are major health problems throughout the world. Globally, nearly 3.5 billion individuals are infected, out of these majorities are children in resource-poor settings [1]. School-age children are of the groups at risk for IPIs due to their frequent contact with soil and less understanding of health standards [2]. Children from developing countries have a high rate of morbidity due to intestinal parasites [3]. Infected children are physically, nutritionally, and cognitively impaired because of the malabsorption of nutrients and, therefore, a reduction in nutritional intake and physical fitness [4]. Besides, intestinal parasites have been associated with stunting growth, physical weakness, and low educational achievement in school [5].

Intestinal parasites are widely distributed in Sub-Saharan Africa largely due to the lower socioeconomic status, poor personal hygiene, unsanitary sewage disposal, inadequate medical care, the inadequacy of safe drinking water supplies, poor nutritional status, and low literacy rate [6]. A very high prevalence of IPIs was reported in Sub-Saharan African countries including in Ghana 15%, in Sudan 56.9%, and in Burkina Faso 84.7% [7–9].

In Ethiopia, IPI is the sixth of the top ten causes of morbidity amongst children [10]. The prevalence is more pronounced due to low quality of drinking water supply and latrine coverage [11]. Several studies in different regions of Ethiopia also supported our view. For instance, an overall prevalence ranging from 22.6% to 62.4% was reported by [12–14]. To get a deeper insight into the magnitude of the problem and design an effective intervention mechanism, more information is required from different localities where similar studies have not been reported. According to health center reports in Maksegnit, 2017-2018, IPIs were the main reason why many people visit health facilities. However, the prevalence of IPIs and associated risk factors were not scientifically documented in the study area. Therefore, the objective of this study was to assess the prevalence of IPIs and associated risk factors in Maksegnit school children.

## 2. Materials and Methods

### 2.1. Study Design and Study Area

A cross-sectional study was conducted from October 2019 to June 2020 among students of Maksegnit Primary School, North Gondor, Ethiopia. The school is located at 37^0^ 45′ 43″ E and 12^o^ 7′ 23″ N-12^0^ 39′ 24″ N and about 748 km far to the North West of the country's capital Addis Ababa. According to the 2007 census of the central statistical agency of Ethiopia, the district has an estimated population of 222,990 [15].

### 2.2. Study Population

School-age children from grades 1 to 8 who were available during the study period, whose parents or guardians signed a written assent form, and willing to participate were included.

### 2.3. Sample Size Determination and Sampling Technique

The desired sample size (*n*) was computed using a single population proportion formula described as *n*=(**Z**^2^ × **P**(1 − **P**))/**d**^2^ [16], where *n* = minimum number of sample size; **Z**^2^=standard value at  **Z**_*α*/2_=1.96,  the *z* table value; *P* = expected prevalence of intestinal parasitic infections in the study area; and *d* = marginal error = 5%, at 95% confidence interval. Since no report was recorded on the prevalence of intestinal parasitic infections in the study area, *P* = 50% was taken. Accordingly, *n*=(1.96)^2^ × 0.5(1 − 0.5)/(0.05)^2^ =384.

Participants were stratified based on their educational level (grade 1 to grade 8). One section quota was then allocated for each grade. The eight sections were selected by the lottery method considering each grade. Proportional sampling was used to determine the number of children to be selected from each section. Finally, the participating children were selected from each section using systematic random sampling techniques by using class roaster as a sample frame.

### 2.4. Data Collection Techniques

A semistructured questionnaire was prepared in English and translated into the local language (Amharic) to collect data on sociodemographic variables, behavioral habits, and environmental factors. Subsequently, cleanliness of students' fingernails, the presence or absence of shoe wearing, and environmental sanitation were observed.

### 2.5. Stool Sample Collection and Processing

The students were requested to bring about 5 g of fresh stool sample in a labeled plastic cup and submit it immediately to the laboratory room. Primarily, the nature of the sample was examined macroscopically. Microscopic examination was done by direct wet mount and formol-ether concentration methods following the procedures in WHO guidelines [17]. A 2 g of stool sample was examined by the direct wet mount technique, and the remaining 3 g was examined by the formol-ether concentration technique. Both x10 and x40 objectives were used for the identification of intestinal parasites. The positivity for the intestinal parasite was confirmed when observed in either of the methods used.

### 2.6. Data Quality Control

To ensure quality control, the questionnaires were pretested by taking samples before the data collection. All samples were labeled with the student's code to avoid confusion of their results. Each questionnaire was checked whether the necessary information was properly filled. Children were interviewed using their local language (Amharic) and standard operating procedures were used for specimen collection and processing.

### 2.7. Data Analysis

Data were coded, stored in Microsoft Excel, and analyzed using SPSS version 20. Univariate logistic regression analysis was used to assess the association between each risk factor and IPIs. To determine the independent risk factors for infection, multiple logistic regression analysis was performed using the adjusted odds ratio at a 95% confidence interval. The result of the association was considered statistically significant when the *P* value was less than 0.05.

## 3. Results

### 3.1. Sociodemographic Characteristics of the Study Participants

A total of 384 students took part in the study, which yielded a 100% response rate. 206 (53.6%) were females, the mean age of the study participants was 12.08 years, 291 (75.8%) of the study participants were urban residents, and 244 (63.5%) of the students' mothers were homemakers (Table 1).

### 3.2. Prevalence of Intestinal Parasitic Infections

The wet mount and formol-ether concentration techniques revealed that five species of intestinal parasites were identified with an overall prevalence of 155 (40.4%). The most prevalent intestinal parasites identified were *Ascaris lumbricoides* 122 (31.8%) and *Entamoeba histolytica* 18 (4.7%), respectively (Table 2). Of 155 positive individuals, 149 (39%) were a single infection and the rest 6 (1.6%) were a double infection. The most prevalent parasite species in single and double infections were “*A. lumbricoides”* and “*A. lumbricoides* plus *E. histolytica”*, respectively.

### 3.3. Factors Associated with Intestinal Parasitic Infection

Univariate analysis model showed that grade level, residence, drinking water source, latrine availability, defecation habit, hand washing habit before a meal, hand washing habit after defecation, cleanliness of fingernails, mothers' education, and pet availability were significantly associated with any IPIs identified (*P* < 0.05) (Table 3). On the other hand, age group, sex, shoe wearing, eating raw meat, eating raw vegetables, and merchant mothers were not significantly associated with IPIs (*P* > 0.05) (Table 3). To determine the independent risk factors, multivariate analysis was performed for all variables that were significantly associated with any IPIs at the univariate analysis.

According to multivariate analysis, students who consume river and well water were 0.234 (CI = 0.069, 0.797; *P* < 0.05) and 0.169 (CI = 0.093, 0.304; *P* ≤ 0.001) times more probable than those who used pipe water to develop IPIs, respectively. Students who did not wash their hands before the meal were 0.128 (CI  = 0.028, 0.591; *P* < 0.05) times more probable to develop IPIs than their counterparts. Students who did not wash their hands after defecation were 0.166 (CI = 0.106, 0.260; *P* ≤ 0.001) times more probable than their counterparts to develop IPIs. Children who defecate on open field are 0.142 (CI = 0.089, 0.22; *P* ≤ 0.001) times more probable than their counterparts to acquire IPIs. Moreover, students who had unclean fingernails were 0.146 (CI = 0.127, 0.308; *P* ≤ 0.001) times more probable than those who had clean fingernails to develop IPIs (Table 3).

## 4. Discussion

The objective of this study was to assess the prevalence and risk factors of IPIs in Maksegnit Primary School children. The overall prevalence of IPIs (40.4%) was higher when compared with earlier studies reported inside the country [18]. The result is also comparable with the number of cases reported in Gamo, Ethiopia [19], and Turkey [4]. In contrast, the prevalence is lower than studies reported inside the country [1, 10, 13, 20] and in other countries, India [21] and Burkina Faso [9]. The discrepancy in the results of the studies may be correlated with the differences in the diagnostic technique, drinking water source, environmental sanitation, and living conditions of the participants.

Among the intestinal parasites identified, *A. lumbricoides* (31.8%) was the dominant one. The observed prevalence of *A. lumbricoides* is greater when compared with studies inside the country [10, 11, 13, 18, 20, 21] and other countries, India [21] and Turkey [4]. However, this is lower than the studies conducted inside the country [22] and outside the country, Cameron [20]. The high prevalence of *Ascaris* infection could be attributed to the fact that the egg of *A. lumbricoides* can live for up to ten years in a warm, shaded, and moist environment, which could be the reason for their long-term infection. *E. histolytica* was detected in 18 (4.7%). This result was in agreement with different researchers inside the country [13]. This result was also lower compared with studies in different regions of Ethiopia [1, 11, 22, 23]. The high prevalence of *E. histolytica* may be due to the consumption of unsafe water by the students.

In this study, the prevalence of *H. nana* was 15 (3.9%). This was relatively higher than studies done in Ethiopia [6, 11, 20, 22], but relatively lower as compared to other studies reported in the country [1, 4, 19]. The high prevalence in this study may be attributed to open field defecation habits of the students and the autoinfection mode of transmission of the parasite [24]. The prevalence of *G. duodenalis* and *S. mansoni* was 0.8%. This was very lower than the study reported in Dembiya (3%) [23] and Teda (12.4%, 8.9%), respectively [25].

In this study, the prevalence of double IPIs was 6 (1.6%). This was much lower than the study reported from Gojjam (14%) [10]. The observed difference in the detection of parasites in the two study localities might be due to the variations in the geographical condition, sample size, and method used.

This study also assessed the possible association of IPIs with potential risk factors. One of the factors strongly associated with IPIs was unclean fingernails (AOR = 0.146; CI = 0.084, 0.256; *P* ≤ 0.001). This finding is supported by previous studies reported in Ethiopia [13]. Students who use river water (AOR = 0.124; *P* < 0.05) were more likely to get exposed to IPIs than their counterparts. This finding is in line with earlier studies in Ethiopia [1, 10, 13]. Poor quality of water could be the source of different intestinal parasites where animals and human wastes could flood into the river or unprotected well.

A significantly higher prevalence of IPIs was also found among students with poor hand washing habits after defecation compared with their counterparts. This is supported by prior studies in Ethiopia [25–27] and elsewhere [20]. Students who defecated on an open field were also (AOR = 0.216; *P* ≤ 0.001) more likely to acquire IPIs as compared to their counterparts. This may be because many of the students defecated on open fields, contributing to pollution of the environments that favor fecal-oral transmission of intestinal parasites in the community.

## 5. Conclusion

The present study showed that Maksegnit Primarily School children are highly infected by intestinal parasites. *A. lumbricoides* and *E histolytica* were the predominant intestinal parasites detected among the students. Factors such as open field defecation habit, poor hand washing after defecation, use of unsafe water sources, and the presence of unclean fingernails were strongly associated with the occurrence of IPIs. Therefore, the provision of education on personal hygiene and environmental sanitation for students and their parents may enhance their awareness of fecal-oral transmissions of intestinal parasites.

### 5.1. Limitations

Both direct wet mount and formol-ether concentration methods were used to identify the parasites. However, due to resource constraints, we did not perform other sensitive methods specific for some intestinal parasites such as PCR, Kato-Katz method, and trichome and modified Ziehl–Neelsen staining methods.

## Figures and Tables

**Table 1:** Sociodemographic characteristics of Maksegnit grade school children, 2018/2019.

Variables	Categories	Frequency	Percent
Grade level	1–4	192	50
	5–8	192	50

Age	7–10	122	31.8
	11–14	185	48.2
	15–18	77	20.1

Sex	Male	178	46.4
	Female	206	53.6

Residence	Urban	291	75.8
	Rural	93	24.2

Mothers' education	Illiterate	222	57.8
	Literate	162	42.2

Mothers' occupation	Housewife	244	63.5
	Merchant	99	25.8
	Daily labor	6	1.6
	Government-employed	35	9.1

**Table 2:** The prevalence of intestinal parasitic infections among Maksegnit grade school children, 2018/2019.

Intestinal parasites	Frequency	Percent
*Protozoans*		
*E. histolytica*	18	4.7
*Giardia duodenalis*	3	0.8

*Helminths*		
*A. lumbricoides*	122	31.8
*Hymenolepis nana*	15	3.9
*Schistosoma mansoni*	3	0.8

*Double infection*		
*A. lumbricoides* _+_ *E. histolytica*	3	0.8
*A. lumbricoides* _+_ *H. nana*	2	0.5
*H. nana* + *S. mansoni*	1	0.3
Single infection	149	39
Double infection	6	1.6
Overall positive infection	155	40.4

**Table 3:** Multivariate logistic regression analysis of potential risk factors associated with intestinal parasitic infections (IPIs) in Maksegnit school children, 2018/2019.

Risk factors	Categories	Intestinal parasites			
		Positive, *n* (%)	Negative, *n* (%)	COR (95% CI)	AOR (95%CI)
Grade level	1–4	88 (56.8)	104 (45.4)	0.633 (0.420–0.955) ^*∗*^	—
	5–8	67 (43.2)	125 (54.6)	1	
Age group	7–10	50 (32.3)	72 (31.4)	0.734 (0.405–1.330)	—
	11–14	79 (51)	106 (46.3)	0.684 (0.393–1.191)	
	15–18	26 (16.8)	51 (22.3)	1	
Sex	Male	76 (49)	102 (44.5)	0.835 (0.555–1.256)	—
	Female	79 (51)	127 (55.5)	1	
Residence	Rural	63 (40.6)	30 (13.1)	0.220 (0.134–0.363) ^*∗*^	—
	Urban	92 (59.4)	199 (86.9)	1	
Source of drinking water	River/stream	8 (5.2)	4 (1.7)	0.234 (0.069–0.797) ^*∗*^	0.124 (0.027–0.569) ^*∗*^
	Well	50 (32.3)	18 (7.9)	0.169 (0.093–0.304) ^*∗*^	0.506 (0.238–1.078)
	Pipe	97 (62.6)	207 (90.4)	1	1
Latrine availability	Yes	76 (49)	201 (87.8)	1	—
	No	79 (51)	28 (12.2)	0.134 (0.081–0.222) ^*∗*^	—
Defecation habit	Open field	95 (61.3)	42 (18.3)	0.142 (0.089–0.226) ^*∗∗*^	0.216 (0.122–0.382) ^*∗∗*^
	Latrine	60 (38.7)	178 (81.7)	1	1
Shoe wearing habit	Sometimes	17 (11)	21 (9.2)	0.820 (0.417–1.609)	—
	Always	138 (89)	208 (90.8)	1	—
Hand washing habit before a meal	Yes	145 (93.5)	227 (99.1)	1	—
	No	10 (6.5)	2 (0.9)	0.128 (0.028–0.591) ^*∗*^	—
Hand washing habit after defecation	Yes	51 (32.9)	171 (74.7)	1	1
	No	104 (67.1)	58 (25.3)	0.166 (0.106–0.260) ^*∗∗*^	0.236 (0.134–0.417) ^*∗∗*^
Eating raw meat	Yes	39 (25.2)	41 (17.9)	0.649 (0.395–1.065)	—
	No	116 (74.8)	118 (82.1)	1	—
Eating raw/uncooked vegetables	Yes	47 (30.3)	51 (22.3)	0.658 (0.414–1.046)	—
	No	108 (69.7)	178 (77.7)	1	—
Dirty fingernails	Present	97 (62.6)	57 (24.9)	0.198 (0.127–0.308) ^*∗∗*^	0.146 (0.084–0.256) ^*∗∗*^
	Absent	58 (37.4)	172 (75.1)	1	1
Mothers' education	Illiterate	118 (76.1)	104 (45.4)	0.261 (0.166–0.410) ^*∗∗*^	—
	Literate	37 (23.9)	125 (54.6)	1	—
Mothers' occupation	Housewife	117 (75.5)	127 (55.5)	0.271 (0.114–0.645) ^*∗*^	—
	Merchant	27 (17.4)	72 (31.4)	0.667 (0.261–1.705)	—
	Daily labour	4 (2.6)	2 (0.9)	0.125 (0.019–0.826) ^*∗*^	—
	Government-employed	7 (4.5)	28 (12.2)	1	—
Pet availability	Yes	91 (58.7)	108 (47.2)	0.628 (0.416–0.948) ^*∗*^	—
	No	64 (41.3)	121 (52.8)	1	—

^*∗*^Statistically significant at *P* < 0.05;  ^*∗∗*^Significant association at *P* < 0.001; COR = crude odds ratio; CI = confidence interval; AO = adjusted odds ratio.

## Data Availability

The datasets used or analyzed during the current study are available from the corresponding author on reasonable request.

## References

[1] Ayalew A., Debebe T., Worku A. (2011). Prevalence and risk factors of intestinal parasites among Delgi school children, North Gondar, Ethiopia. *Journal of Parasitology and Vector Biology*.

[2] Barazesh A., Fouladvand M., Tahmasebi R., Heydari A., Kooshesh F. (2016). Prevalence of intestinal parasitic infections among primary school children in Bushehr, Iran. *Avicenna Journal of Clinical Microbiology and Infection*.

[3] Stephenson L. S., Latham M. C., Ottesen E. A. (2001). Malnutrition and parasitic helminth infections. *Parasitology*.

[4] Doni N. Y., Gurses G., Simsek Z., Zeyrek F. Y. (2015). Prevalence and associated risk factors of intestial parasites among children of farm workers in the southeastern Anatolian region of Turkey. *Annals of Agricultural and Environmental Medicine*.

[5] Legesse M., Erko B. (2004). Prevalence of intestinal parasites among schoolchildren in a rural area close to the southeast of Lake Langano, Ethiopia. *The Ethiopian Journal of Health Development (EJHD)*.

[6] Gebretsadik G. (2016). Prevalence of intestinal Parasites and associated risk factors among schoolchildren of Homesha District (Woreda) in Benishangul-Gumuz regional State, western Ethiopia. *Journal of Family Medicine and Health Care*.

[7] Forson A. O., Arthur I., Olu-Taiwo M., Glover K. K., Pappoe-Ashong P. J., Ayeh-Kumi P. F. (2017). Intestinal parasitic infections and risk factors: a cross-sectional survey of some school children in a suburb in Accra, Ghana. *BMC Research Notes*.

[8] Suliman M. A., Magboul A. M., Mohammed H. Y. (2019). Prevalence of intestinal parasitic infections and associated risk factors among school children in white Nile state, Sudan. *Journal of Infectious Disease and Diagnosis*.

[9] Erismann S., Diagbouga S., Odermatt P. (2016). Prevalence of intestinal parasitic infections and associated risk factors among schoolchildren in the Plateau Central and Centre-Ouest regions of Burkina Faso. *Parasites & Vectors*.

[10] Workneh T., Esmael A., Ayichiluhm M. (2014). Prevalence of intestinal parasitic infections and associated factors among debre elias primary schools children, east Gojjam zone, Amhara region, North west Ethiopia. *Journal of Bacteriology and Parasitology*.

[11] Gebru A. A., Tamene B. A., Bizuneh A. D. (2015). Prevalence of intestinal parasites and associated risk factors at red cross clinic and Chelaleki health center, East Wollega Zone, Ethiopia. *Science Journal of Public Health*.

[12] Sisay A., Lemma B. (2019). Assessment on the prevalence and risk factors of gastrointestinal parasites on schoolchildren at Bochesa Elementary School, around Lake Zwai, Ethiopia. *BMC Research Notes*.

[13] Maru D. S. (2015). *P*revalence of intestinal parasitic infections and associated risk factors among school children in Adigrat town, northern Ethiopia. *International Journal of Emerging Trends in Science and Technology*.

[14] Sitotaw B., Shiferaw W. (2020). Prevalence of intestinal parasitic infections and associated risk factors among the first-cycle primary schoolchildren in sasiga district, southwest Ethiopia. *Journal of Parasitology Research*.

[15] CSA (2016). *The 2015/16 Ethiopian Household Consumption–Expenditure (Hce Survey Statistical Report Result For Amhara Region.Pdf)*.

[16] Daniel W. W., Cross C. L. (2018). *Biostatistics: A Foundation for Analysis in the Health Sciences*.

[17] WHO (1991). *Basic Laboratory Methods in Medical Parasitology*.

[18] Zemene T., Shiferaw M. B. (2018). Prevalence of intestinal parasitic infections in children under the age of 5 years attending the Debre Birhan referral hospital, North Shoa, Ethiopia. *BMC Research Notes*.

[19] Wegayehu T., Tsalla T., Seifu B., Teklu T. (2013). Prevalence of intestinal parasitic infections among highland and lowland dwellers in Gamo area, South Ethiopia. *BMC Public Health*.

[20] Kwenti T. E., Nkume F. A., Tanjeko A. T., Kwenti T. D. B. (2016). The effect of intestinal parasitic infection on the clinical outcome of malaria in coinfected children in Cameroon. *PLoS Neglected Tropical Diseases*.

[21] Ashok R., Suguneswari G., Satish K., Kesavaram V. (2013). Prevalence of intestinal parasitic infection in school going children in Amalapuram, Andhra Pradesh, India. *Shiraz E-Medical Journal*.

[22] Mengistu A., Gebre-Selassie S., Kassa T. (2007). Prevalence of intestinal parasitic infections among urban dwellers in southwest Ethiopia. *Ethiopian Journal of Health Development*.

[23] Gizaw Z., Adane T., Azanaw J., Addisu A., Haile D. (2018). Childhood intestinal parasitic infection and sanitation predictors in rural Dembiya, northwest Ethiopia. *Environmental Health and Preventive Medicine*.

[24] Jemaneh L. (2000). The epidemiology of schistosomiasis mansoni and soil-transmitted helminths in elementary school children from the South Gondar Zone of the Amhara National Regional State, Ethiopia. *Ethiopian Medical Journal*.

[25] Abate A., Kibret B., Bekalu E. (2013). Cross-sectional study on the prevalence of intestinal parasites and associated risk factors in Teda health centre, northwest Ethiopia. *ISRN Parasitology*.

[26] Mama M., Alemu G. (2015). Prevalence and factors associated with intestinal parasitic infections among food handlers of Southern Ethiopia: cross sectional study. *BMC Public Health*.

[27] Teshale T., Belay S., Tadesse D., Awala A., Teklay G. (2018). Prevalence of intestinal helminths and associated factors among school children of Medebay Zana wereda; North Western Tigray, Ethiopia 2017. *BMC Research Notes*.

